# Association of Child Placement in Out-of-Home Care With Trajectories of Hospitalization Because of Suicide Attempts From Early to Late Adulthood

**DOI:** 10.1001/jamanetworkopen.2020.6639

**Published:** 2020-06-02

**Authors:** Ylva B. Almquist, Yerko Rojas, Bo Vinnerljung, Lars Brännström

**Affiliations:** 1 Department of Public Health Sciences, Stockholm University, Stockholm, Sweden; 2 Södertörn University School of Social Sciences, Huddinge, Sweden; 3 Department of Social Work, Stockholm University, Stockholm, Sweden

## Abstract

**Importance:**

Children placed in out-of-home care (OHC) have higher rates of suicidal behaviors, including suicide attempts, compared with those who grow up in their family of origin. Several studies have shown that this elevated risk persists into young adulthood. Yet, our knowledge about any longer-term associations of OHC with suicide attempts is limited.

**Objective:**

To examine how childhood experiences of placement in OHC are associated with trajectories of hospitalization because of suicide attempts (HSA) from early into late adulthood.

**Design, Setting, and Participants:**

This prospective birth cohort study that was conducted in Stockholm, Sweden, and analyzed in March 2020 included 14 559 individuals born in 1953 who were living in the greater metropolitan of Stockholm in November 1963 and followed through registers up until December 2016.

**Exposures:**

Childhood experiences of OHC based on information from the Social Register (age 0-19 years).

**Main Outcomes and Measures:**

Hospitalization because of suicide attempts based on in-patient care data from the National Patient Register. Group-based trajectory modeling was used to cluster individuals according to their probabilities of HSA across adulthood (age 20-63 years).

**Results:**

In this cohort of 14 559 individuals (7146 women [49.1%]), 1320 individuals (9.1%) had childhood experiences of OHC, whereas 525 individuals ( 3.6%) had HSA. A Cox regression analysis showed a substantially higher risk of HSA among those with childhood experiences of OHC (hazard ratio, 3.58; 95% CI, 2.93-4.36) and after adjusting for a range of adverse childhood living conditions (hazard ratio, 2.51; 95% CI, 2.00-3.15). Those with at least 1 HSA were grouped into 4 trajectories: (1) peak in middle adulthood (66 [12.6%]), (2) stable low across adulthood (167 [31.8%]), (3) peak in early adulthood (210 [40.0%]), and (4) peak in emerging adulthood (82 [15.6%]). A multinomial regression analysis suggested that those with experiences of OHC had higher risks of following any of these trajectories (trajectory 1: relative risk ratio [RRR], 2.91; 95% CI, 1.61-5.26; trajectory 2: RRR, 3.18; 95% CI, 2.21-4.59; trajectory 3: RRR, 4.32; 95% CI, 3.18-5.86; trajectory 4: RRR, 3.26; 95% CI, 1.94-5.46). The estimates were reduced after adjusting for adverse childhood living conditions.

**Conclusions and Relevance:**

The findings suggest that the elevated risk of suicide attempts among former child welfare clients does not cease after young adulthood, indicating the necessity for clinical attention to childhood experiences of OHC as a risk marker for suicidal behavior across the life span.

## Key Points

**Question:**

How are childhood experiences of placement in out-of-home care associated with trajectories of hospitalization because of suicide attempts from early into late adulthood?

**Findings:**

In this cohort study of 14 559 individuals, individuals were grouped into 4 trajectories with differential onset of suicide attempts across adulthood. Childhood experiences of placement in out-of-home care were associated with increased risks of following each of these trajectories.

**Meaning:**

The elevated risk of suicide attempts among former child welfare clients persists into young adulthood, indicating the necessity for clinical attention to childhood experiences of out-of-home care as a risk marker for suicidal behavior across the life span.

## Teaser

This cohort study examines the association between childhood experiences of placement in out-of-home care and trajectories of hospitalization because of suicide attempts from early into late adulthood in Sweden.

## Introduction

Suicidal behaviors among children and young people have been identified as a major public health problem.^1^ The prevalence of such behaviors is particularly prominent among individuals placed in foster family or residential care (out-of-home care [OHC]). For example, a recent systematic review and meta-analysis showed that children and young people in OHC are more than 3 times more likely to attempt suicide compared with noncare populations.^2^ A few prospective studies have demonstrated that this difference does not cease after exiting care but rather extends further into young adulthood.^3,4^ Whether the same holds true for later phases in life remains to be explored. There are good reasons to expect this to be the case, considering that the elevated risks of mental and behavioral disorders among individuals with childhood experiences of placement in OHC have been found to last well into midlife.^5,6,7^ We also find support for this notion in the research focusing on adverse childhood experiences (ACEs), a concept that overlaps with experiences of OHC in theory and practice, and lifetime suicide attempts.^8,9,10,11^ Yet, with some notable exceptions,^12^ most studies have based their measurements of ACE on retrospective data, which are known to be sensitive for recall bias.^13^

Rates of suicidal behaviors differ across age groups and the transition from adolescence to early adulthood is generally when the prevalence of suicide attempts peaks.^14^ However, past research has seldom accounted for the fact that there is also considerable individual variability in developmental trajectories of suicidal behaviors^10,15^: whereas some individuals attempt suicide only once, others do repeated attempts.^14^ This is notable given that the latter group may reflect the phenomena of chronic suicidality, the idea that there are individuals with more or less constant suicidal thoughts who tend to engage in repetitive self-destructive patterns of behavior,^16^ which has been particularly associated with ACE.^17^ Among the studies that do examine the prospective, longitudinal development of suicidal behaviors, most are based on small-scale, clinical samples with relatively short-term follow-ups limited to adolescence and/or early adulthood.^14,18^ It has nevertheless been suggested that suicidal behaviors at earlier ages in the life course may differ in important ways from suicidal behaviors occurring in later life, not least in terms of etiology.^19^ Thus, if we could identify common trajectories of suicide attempts spanning all of adulthood, it is plausible that these would be differentially associated with OHC depending on their onset.^10^ This can inform practitioners and policy makers aiming to improve the life chances of children and young people for whom society, acting in loco parentis, has assumed responsibility.

Based on a prospective study of a 1953 cohort from Stockholm, Sweden (n = 14 608), this study attempts to fill the previously identified research gaps by not only exploring how childhood experiences of placement in OHC (age 0-19 years) are associated with the risk of hospitalization because of suicide attempts (HSA) from early into late adulthood (age 20-63 years), but also to group the individuals into trajectories of suicide attempts and determine their association with OHC. Moreover, following the previous literature focusing on suicidal behaviors in OHC populations, we will include possibly explanatory factors reflecting adverse childhood living conditions.^3,4^

## Methods

### Data Material

Data were drawn from the Stockholm Birth Cohort Multigenerational Study (SBC Multigen), a prospective cohort study established in 2018/2019 through a probability matching of 2 anonymized, longitudinal data materials: the Stockholm Metropolitan Study (SMS) and the RELINK53 project. The SMS is defined as all individuals born in 1953 who were living in the Stockholm metropolitan area in 1963 (n = 15 117). Survey and register data were collected until December 1986, after which the SMS was deidentified. RELINK53 is defined as everyone born in 1953 and residing in Sweden in 1960, 1965, and/or 1968, as well as their family linkages, comprising administrative register information between December 1953 and December 2018 for a total of 2 390 753 individuals. Using an algorithm based on variables identical to both data materials, 14 608 cohort members from the SMS were matched with RELINK53 and thus included in the SBC Multigen.^20^ The Stockholm regional ethical review board approved the creation of the SBC Multigen. The need for consent was waived because of the anonymized nature of the data material used. The current study follows the Strengthening the Reporting of Observational Studies in Epidemiology (STROBE) reporting guidelines for cohort studies.

### Variables

Childhood experiences of placement in OHC (1953-1972; ages 0-19 years) was the main independent variable in this study. This information was drawn from manually collected data from the social registers kept by the municipalities in the Stockholm region. Compared with many other countries, Swedish legislation has for many decades had an emphasis on child welfare, a belief in the legitimacy of state interventions into family life, and an expanded view of perceived child protection jurisdiction.^21^ This was also the case in the 1960s and 1970s, resulting in relatively high rates of OHC in this cohort (1320 [9.1%]).

**Figure 1.**
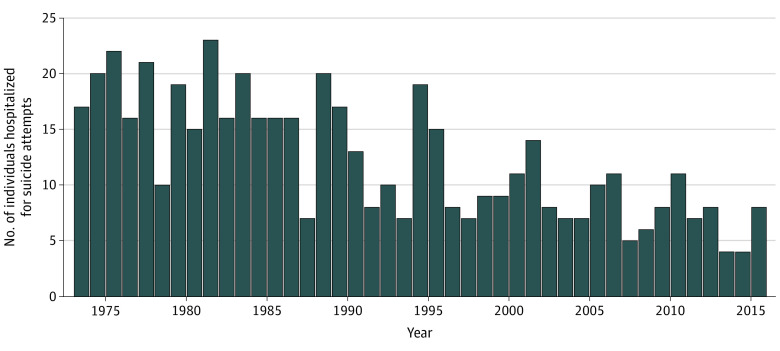
Number of Individuals Hospitalized Because of Suicide Attempts per Year From 1973 to 2016

Hospitalization because of suicide attempts (1973-2016; ages 20-63 years) constituted the dependent variable. It was constructed based on information from the National Patient Register. This register contains records of in-patient care (overnight stays at the hospital), classified according to the 8th (1973-1986), 9th (1987-1996), and 10th (1997-2016) revisions of the* International Classification of Disease* (*ICD*). Following recommendations made in previous studies,^3,4,22,23^ suicide attempts were indicated through diagnoses on intentional self-harm (*ICD 8/9*: E950-E959; *ICD 10*: X60-X84) and events of undetermined intent (*ICD 8/9*: E980-E989; *ICD 10*: Y10-Y34). We first created a binary indicator for the entire period, showing that 525 (3.6%) of the cohort members had at least 1 HSA during the follow-up. Next, we constructed a binary indicator of HSA per year (Figure 1). Among those who died during the follow-up (1283 [8.8%]), missing values were assigned to any subsequent indicators.

Apart from sex, we included 7 covariates reflecting adverse childhood living conditions based on register information: socioeconomic position, educational level, poverty, death, criminality, alcohol use, and mental health problems. These variables have been shown to be associated with the risk of OHC in several previous studies.^24,25,26,27^ Additional information for the study variables, including descriptive statistics, are shown in Table 1 and eTable 1 in the Supplement.

**Table 1.** Descriptive Statistics for the Study Variables for 14 559 Individuals

Variables	Period	Age, y	Data source(s)	No. (%)
Hospitalization because of suicide attempts				
No	1973-2016	20-63	National Patient Register	14 034 (96.4)
Yes				525 (3.6)
Out-of-home care				
No	1953-1972	0-19	Social Register	13 239 (90.9)
Yes				1320 (9.1)
Sex				
Men	1953	0	Medical Birth Register	7413 (50.9)
Women				7146 (49.1)
Socioeconomic position^a^				
Low	1953	0	Occupational registers	7217 (49.6)
High				7342 (50.4)
Educational level^b^				
Low	1960	7	1960 Census	10 807 (74.2)
High				3752 (25.8)
Poverty^c^				
No	1953-1972	0-19	Social Register	12 114 (83.2)
Yes				2445 (16.8)
Death^d^				
No	1953-1972	0-19	Social Register/Register of the total population	13 650 (93.8)
Yes				909 (6.2)
Criminality^e^				
No	1953-1972	0-19	National Crime Register	13 414 (92.1)
Yes				1145 (7.9)
Alcohol use^f^				
No	1953-1972	0-19	Social Register	13 657 (93.8)
Yes				902 (6.2)
Mental health problems^g^				
No	1953-1972	0-19	Social Register	13 527 (93.6)
Yes				932 (6.4)

^a^ The socioeconomic position of the head of household, which in most cases was the father. Here, middle and upper-middle positions were classified as high and all others (middle to low position unclassified, and missing) were collapsed into low.

^b^ The number of members of the household with a degree from upper secondary school. The presence of at least 1 household member with a degree was identified as high and the remaining (including missing) were identified as low.

^c^ Coded as yes if the parents had any records of receiving social welfare.

^d^ Coded as yes if there were any records of the father or mother having died.

^e^ Only available for the father and coded as yes if there were any records of crime.

^f^ Coded as yes if the parents had been registered for alcoholism or incidents of drunkenness.

^g^ Coded as yes if there were any records referring to the father’s or mother’s psychiatric problems, receipt of psychiatric care, or suicide.

### Statistical Analysis

Those who died before the start of the follow-up (ie, before January 1, 1973) were excluded (n = 49), resulting in a sample consisting of 14 559 individuals. Three types of statistical analysis were performed in Stata, version 14.0 (StataCorp). First, a Cox regression analysis (stcox command) was used to determine the association between childhood experiences of OHC and HSA in adulthood, producing hazard ratios (HRs) with 95% confidence intervals. This approach made it possible to take time under risk into account. Individuals entered the study on January 1, 1973, and were censored in the event of death, on the date of their first HSA, or at the end of follow-up (December 31, 2016). Two models were generated: one with unadjusted estimates and one that was fully adjusted.

Second, we applied group-based trajectory modeling (GBTM) to group individuals with at least 1 HSA (525 [3.6%]) into trajectories using the traj plugin.^28^ GBTM is a specialized type of finite (discrete) mixture models.^28^ It has been frequently applied in previous research as a way of examining developmental trajectories based on repeated measurements of a specific outcome over age or time.^29^ The model parameters are derived from maximum likelihood estimation, after which the optimal number of groups and the appropriate degree of the polynomial are determined. For each group, the model estimates an individual’s probability to belong to that group; group membership is subsequently determined based on the group for which the individual shows the highest probability. Model selection is an intricate task and there is no consensus regarding the choice of criteria. For the goal of balancing comprehensibility and use with an adequate exploration of complexity, it has been suggested that several principles should be combined.^28^ In this study, a logit model with 4 trajectories and a cubic polynomial function was preferred (eTables 2 and 3 in the Supplement).

Third, a multinomial regression analysis (mlogit command) was used to investigate the association between OHC and the trajectories of HSA, producing relative risk ratios (RRRs) with 95% confidence intervals. Here, all individuals without any HSA served as the reference group. The results thus show the relative risk among those with childhood experiences of OHC vs those without any such experiences to follow the 4 trajectories of HSA as opposed to being in the reference group. Similar to the procedure for the Cox regression analysis, 2 models were generated. Statistical significance was set at *P *< .05.

## Results

The results regarding childhood experiences of OHC and the risk of HSA in adulthood are presented in Table 2. The unadjusted model shows that the hazard among individuals who experienced OHC is 3.58 times higher (95% CI, 2.93-4.36) compared with those without such experiences. Apart from sex, all other covariates are associated with HSA. While the association between OHC and HSA becomes reduced in the adjusted model, it remains strong and statistically significant (HR, 2.51; 95% CI, 2.00-3.15).

**Table 2.** Childhood Experiences of Out-of-Home Care From Age 0 to 19 Years and the Risk of Being Hospitalized Because of Suicide Attempts in Adulthood From Age 20 to 63 Years

Variables	Hospitalization because of suicide attempts, HR (95% CI)	
	Unadjusted model	Adjusted model^a^
Out-of-home care		
Yes	3.58 (2.93-4.36)	2.51 (2.00-3.15)
No	1 [Reference]	1 [Reference]
Sex		
Women	1.04 (0.87-1.23)	1.06 (0.89-1.25)
Men	1 [Reference]	1 [Reference]
Socioeconomic position		
High	0.72 (0.60-0.85)	0.88 (0.72-1.07)
Low	1 [Reference]	1 [Reference]
Educational level		
High	0.71 (0.58-0.88)	1.00 (0.79-1.29)
Low	1 [Reference]	1 [Reference]
Poverty		
Yes	2.45 (2.04-2.94)	1.42 (1.12-1.79)
No	1 [Reference]	1 [Reference]
Death		
Yes	1.42 (1.05-1.93)	1.11 (0.82-1.51)
No	1 [Reference]	1 [Reference]
Criminality		
Yes	2.10 (1.65-2.67)	1.26 (0.96-1.66)
No	1 [Reference]	1 [Reference]
Alcohol use		
Yes	2.47 (1.92-3.17)	1.25 (0.94-1.68)
No	1 [Reference]	1 [Reference]
Mental health problems		
Yes	2.66 (2.09-3.39)	1.35 (1.02-1.77)
No	1 [Reference]	1 [Reference]

Abbreviation: HR, hazard ratio.

^a^ Adjusted for out-of-home care, sex, socioeconomic position, educational level, poverty, death, criminality, alcohol use, and mental health problems.

**Figure 2.**
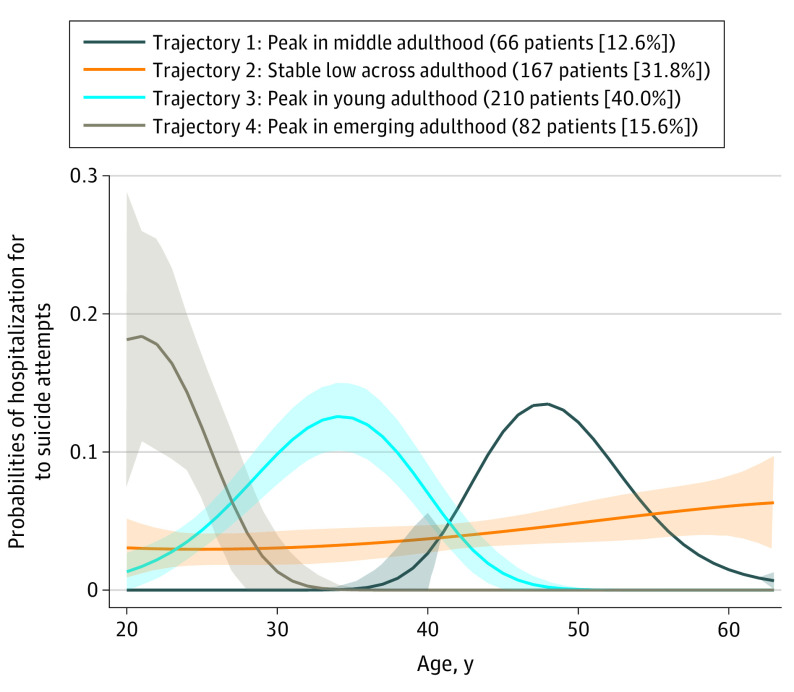
Trajectories of Hospitalization Because of Suicide Attempts for 525 Individuals Results from group-based trajectory modeling. The colored areas illustrate 95% confidence intervals.

Figure 2 demonstrates the results from GBTM in which the individuals were categorized into 4 trajectories of HSA. The largest group (210 [40.0%]) is characterized by a peak in the probability of HSA in early adulthood, starting around age 25 years and ending around age 40 years (trajectory 3). Individuals in the second largest group (167 [31.8%]) follow a trajectory with stable probabilities across the span of adulthood (trajectory 2), although there is indication of slightly increasing probabilities toward the end of the follow-up. The third largest group (82 [15.6%]) is characterized by a peak in emerging adulthood (trajectory 4): the individuals in this group display relatively high probabilities of HSA at the beginning of the follow-up and then rapidly decreasing probabilities that reach 0 around age 30 years. Finally, the smallest group (66 [12.6%]) consists of individuals who follow a trajectory that peaks in middle adulthood, which spans ages 40 to 55 years (trajectory 1).

The results from multinomial regression analysis are shown in Table 3. In the unadjusted model, individuals with childhood experiences of OHC consistently show higher risks of following trajectories characterized by HSA compared with individuals without any such experiences. More specifically, their risk of being in trajectory 1 (peak in middle adulthood) is almost 3-fold (RRR, 2.91; 95% CI, 1.61-5.26), more than 3-fold for trajectory 2 (stable low across adulthood; RRR, 3.18; 95% CI, 2.21-4.59) and trajectory 4 (peak in emerging adulthood; RRR, 3.26; 95% CI, 1.94-5.46), and more than 4-fold for trajectory 3 (peak in young adulthood; RRR, 4.32; 95% CI, 3.18-5.86). In the adjusted model, the estimates for OHC are reduced, but individuals with experiences of OHC still have more than a 2-fold risk of being in any of the 4 trajectories (RRR, 2.23-2.79).

**Table 3.** Association Between Childhood Experiences of Out-of-Home Care From Ages 0 to 19 Years and Trajectories of Hospitalization Because of Suicide Attempts From Early Into Late Adulthood From Ages 20 to 63 Years

Variables	Trajectories of hospitalization because of suicide attempts, RRR (95% CI)^a^^,^^b^			
	Trajectory 1^c^	Trajectory 2^c^	Trajectory 3^c^	Trajectory 4^c^
**Unadjusted model**				
Out-of-home care				
Yes	2.91 (1.61-5.26)	3.18 (2.21-4.59)	4.32 (3.18-5.86)	3.26 (1.94-5.46)
No	1 [Reference]	1 [Reference]	1 [Reference]	1 [Reference]
Sex				
Women	1.33 (0.81-2.16)	1.13 (0.83-1.53)	0.86 (0.65-1.13)	1.26 (0.82-1.96)
Men	1 [Reference]	1 [Reference]	1 [Reference]	1 [Reference]
Socioeconomic position				
High	0.81 (0.50-1.32)	0.75 (0.55-1.03)	0.74 (0.56-0.98)	0.53 (0.34-0.84)
Low	1 [Reference]	1 [Reference]	1 [Reference]	1 [Reference]
Educational level				
High	0.70 (0.38-1.28)	0.65 (0.44-0.96)	0.78 (0.56-1.08)	0.69 (0.40-1.19)
Low	1 [Reference]	1 [Reference]	1 [Reference]	1 [Reference]
Poverty				
Yes	2.25 (1.33-3.81)	2.21 (1.58-3.09)	2.93 (2.20-3.99)	2.14 (1.33-3.45)
No	1 [Reference]	1 [Reference]	1 [Reference]	1 [Reference]
Death				
Yes	1.25 (0.50-3.12)	1.29 (0.73-2.28)	1.52 (0.94-2.44)	1.65 (0.79-3.43)
No	1 [Reference]	1 [Reference]	1 [Reference]	1 [Reference]
Criminality				
Yes	2.17 (1.10-4.27)	2.14 (1.39-3.29)	2.27 (1.55-3.30)	1.69 (0.87-3.28)
No	1 [Reference]	1 [Reference]	1 [Reference]	1 [Reference]
Alcohol use				
Yes	1.03 (0.37-2.82)	2.04 (1.26-3.31)	3.18 (2.20-4.60)	2.99 (1.65-5.44)
No	1 [Reference]	1 [Reference]	1 [Reference]	1 [Reference]
Mental health problems				
Yes	2.13 (1.01-4.47)	2.10 (1.31-3.37)	3.30 (2.30-4.74)	2.91 (1.60-5.28)
No	1 [Reference]	1 [Reference]	1 [Reference]	1 [Reference]
**Adjusted model**^d^				
Out-of-home care				
Yes	2.25 (1.15-4.42)	2.42 (1.59-3.67)	2.79 (1.96-3.97)	2.23 (1.23-4.04)
No	1 [Reference]	1 [Reference]	1 [Reference]	1 [Reference]
Sex				
Women	1.34 (0.82-2.18)	1.15 (0.84-1.56)	0.88 (0.67-1.16)	1.30 (0.84-2.02)
Men	1 [Reference]	1 [Reference]	1 [Reference]	1 [Reference]
Socioeconomic position				
High	1.02 (0.59-1.76)	0.96 (0.68-1.36)	0.91 (0.66-1.24)	0.58 (0.34-0.99)
Low	1 [Reference]	1 [Reference]	1 [Reference]	1 [Reference]
Educational level				
High	0.87 (0.43-1.73)	0.84 (0.54-1.30)	1.18 (0.80-1.74)	1.17 (0.62-2.23)
Low	1 [Reference]	1 [Reference]	1 [Reference]	1 [Reference]
Poverty				
Yes	1.63 (0.84-3.13)	1.39 (0.91-2.11)	1.59 (1.10-2.30)	1.09 (0.59-2.00)
No	1 [Reference]	1 [Reference]	1 [Reference]	1 [Reference]
Death				
Yes	1.04 (0.41-2.61)	1.05 (0.59-1.86)	1.12 (0.69-1.82)	1.32 (0.63-2.78)
No	1 [Reference]	1 [Reference]	1 [Reference]	1 [Reference]
Criminality				
Yes	1.75 (0.83-3.68)	1.45 (0.90-2.35)	1.22 (0.79-1.87)	0.92 (0.44-1.93)
No	1 [Reference]	1 [Reference]	1 [Reference]	1 [Reference]
Alcohol use				
Yes	0.46 (0.15-1.36)	1.05 (0.61-1.83)	1.51 (0.97-2.33)	1.86 (0.92-3.75)
No	1 [Reference]	1 [Reference]	1 [Reference]	1 [Reference]
Mental health problems				
Yes	1.21 (0.53-2.78)	1.11 (0.65-1.88)	1.48 (0.98-2.24)	1.64 (0.82-3.27)
No	1 [Reference]	1 [Reference]	1 [Reference]	1 [Reference]

Abbreviation: RRR, relative risk ratio.

^a^ Results from multinomial regression analysis, presented as RRR with 95% confidence intervals (N = 14 559).

^b^ Reference group for the outcome includes individuals without any hospitalization because of suicide attempts (RRR = 1.00).

^c^ Trajectory 1: peak in middle adulthood; trajectory 2: stable low across adulthood; trajectory 3: peak in young adulthood; and trajectory 4: peak in emerging adulthood.

^d^ Mutual adjustment for out-of-home care, sex, socioeconomic position, educational level, poverty, death, criminality, alcohol use, and mental health problems.

## Discussion

Previous studies have demonstrated that child welfare populations have elevated risks for suicide attempts that extend into young adulthood.^3,4^ This study expands this knowledge by demonstrating that these risks remain throughout midlife and into late adulthood.

When accounting for heterogeneity in the developmental patterning of suicide attempts, we were able to identify 4 trajectories among individuals with at least 1 HSA: peak in middle adulthood, stable low across adulthood, peak in young adulthood, and peak in emerging adulthood. These findings align with contemporary theories on suicidal behavior.^30^ These theories, mostly based on diathesis stress models, maintain that negative effects of underlying vulnerabilities may be activated by stress. Potentially stressful transitions in life may include aging out of adolescence and leaving school (emerging adulthood), attempting to establishing oneself in the labor market and starting a family of one’s own (young adulthood), and having one’s children leave the family household (middle adulthood).^31,32,33^ This could explain why 3 of the trajectories show peaks around these life phases. The fourth trajectory, demonstrating stable probabilities of suicide attempts across the entire span of adulthood (although slightly increasing toward the end) aligns with the notion of chronic suicidality. Many individuals assigned to this trajectory had at least 2 hospitalizations across the follow-up period (eFigure in the Supplement), which might suggest the presence of some underlying mental health problems or disorders. Chronic suicidal behavior has been suggested to occur particularly among individuals with borderline personality disorder.^16^

Individuals with childhood experiences of placement in OHC were more likely to follow any of the 4 trajectories of suicide attempts compared with being in the reference group (ie, the group without HSA). In contrast to what we initially hypothesized, the association did not vary in any systematic way according to the onset of the trajectories. This could be viewed in light of the diathesis stress perspective. Children in OHC have been identified as belonging to one of the most vulnerable groups in society and are at risk of experiencing various adversities at each subsequent stage of the life course.^34^ Accordingly, their underlying vulnerabilities could be activated by stress occurring at any life stage, including stages much later in life.

Although this study did not focus on the mechanisms behind the association between OHC and HSA, only a limited part of the association was explained by the studied aspects of adverse childhood living conditions. This may suggest that reasons for placement not captured by these indicators, such as child abuse and neglect or the children’s own behavioral problems (including self-destructive behavior), could drive the association. In addition, being removed from the family and placed in societal care may in itself have posed a trauma for the child,^35^ as could experiences of inferior care,^36^ with important consequences for suicidal behavior. If this were the case, the heterogeneity in the developmental patterning of suicide attempts found in this study supports the notion that the association between childhood trauma and subsequent suicidality is not merely reducible to a question of whether one develops a psychopathology. This is important because it implies that screening and prevention efforts should be targeted at individuals with a history of childhood trauma regardless of the presence of psychopathology.^37^

### Strengths and Limitations

A major strength of this study was the use of prospective, longitudinal data derived from administrative registers, which avoided biases associated with retrospective measurements and self-reported information. Another advantage was the relatively large sample size, which not only enabled us to study the highly vulnerable population of children in OHC but also made it possible to retain sufficient statistical power to analyze an outcome as rare as suicide attempts. Moreover, the detailed data available in the SBC Multigen offered the opportunity to investigate a set of indicators reflecting some types of adverse childhood living conditions.

Some important limitations should nonetheless be addressed. For example, we constructed a crude measure of childhood experiences of OHC. Children in care do not constitute a homogenous group; they differ on reasons for placement, age at first placement, number of placements, and type of placement. Previous investigations into long-term risks of morbidity and mortality have shown that placement instability and, in particular, teenage placements due to own behavioral placements, are associated with even worse outcomes.^38^ However, we did not have sufficiently detailed data to capture this heterogeneity in this study. The noncare population is also diverse. For example, in the SBC Multigen, several children came to the attention of the child welfare services but remained in their families (either classified as cases ad acta or receiving in-home services). Similar to placed children, although at lower levels, these children also display elevated risks of poor health outcomes in adulthood compared with those who were never in contact with child welfare services.^24^ While this complexity is important to consider when interpreting this study’s results, it was outside the scope of the study to disentangle these aspects.

Although this study is framed around the concept of suicidal behavior, our empirical analysis was limited to suicide attempts. Several studies have shown that suicidal ideation is a predictor of suicide attempts and, similarly, suicide attempt is a well-established predictor of suicide.^39^ None of this complexity was accounted for in our trajectory models. The SBC Multigen does not contain measures of suicidal ideation, but it has information about cause-specific mortality. While group-based trajectory modeling is not equipped to handle survival data, we could nevertheless confirm that suicide was more common among those who had attempted suicide and particularly for individuals following trajectories 1 and 3. On the other hand, these results also show that only one-third of the suicides were preceded by suicide attempts (eTable 4 in the Supplement).

Another limitation is that we used inpatient care data from the National Patient Register to capture suicide attempts. While this register is generally considered to be high quality,^40^ it does not account for cases of attempted suicides that either did not come to the attention of the health care system or did not require overnight stays at the hospital (ie, outpatient care). We are thus only capturing the most severe cases of suicide attempts. Yet, it should be highlighted that the lifetime prevalence of suicide attempts in our study is comparable with previous studies based on survey data.^14,41^

## Conclusions

The findings suggest that the elevated risk of suicide attempts among former child welfare clients does not cease after early adulthood. This has some salient implications for clinical practice. Hospitalization because of suicide attempt was most prevalent in the transit phase to adulthood, indicating a need of systematic screening for suicidal ideation among the large group that age out of OHC.^42^ Moreover, the transition from child/youth mental health services to adult mental health agencies is lined with pitfalls^43^ that could be avoided with better planning from child welfare agencies. Finally, since the elevated risk of HSA is persistent over the life span, OHC should be viewed as a risk marker for possible suicidal behavior, especially in some subgroups, such as adults with a disability pension.^44^ If this is disseminated to and acted on by health care professionals, it has the potential to reduce rates of suicidal behavior in this vulnerable population.^45,46^

## Notes

Supplement. **eTable 1.** Percentage Distribution and Mean Number of Hospitalizations Among Individuals With at Least One Hospitalization Due to Suicide Attempts, Across the Study Variables and Trajectories **eTable 2.** Model Fit Statistics for the Group-Based Trajectory Modelling (n=525) **eTable 3.** Fitted Coefficients (Maximum Likelihood Estimates) for the Preferred Model (Logit Model With Four Trajectories and a Cubic Polynomial Function), Derived From Group-Based Trajectory Modelling (n=525) **eTable 4.** Distribution of Suicide Across Trajectories of Hospitalization Due to Suicide Attempts (n=14,559) **eFigure.** Distribution (%) of the Number of Hospitalizations Due to Suicide Attempts Across the Trajectories (n=525)

## References

[1] World Health Organization Public health action for the prevention of suicide: a framework. Accessed March 20, 2020. https://apps.who.int/iris/bitstream/handle/10665/75166/9789241503570_eng.pdf;jsessionid=F376095F646AA0C8893764638374B649?sequence=1

[2] Evans R, White J, Turley R, Comparison of suicidal ideation, suicide attempt and suicide in children and young people in care and non-care populations: systematic review and meta-analysis of prevalence. Child Youth Serv Rev. 2017;82:122-129. doi:10.1016/j.childyouth.2017.09.020

[3] Vinnerljung B, Hjern A, Lindblad F Suicide attempts and severe psychiatric morbidity among former child welfare clients--a national cohort study. J Child Psychol Psychiatry. 2006;47(7):723-733. doi:10.1111/j.1469-7610.2005.01530.x 16790007

[4] Björkenstam C, Björkenstam E, Ljung R, Vinnerljung B, Tuvblad C Suicidal behavior among delinquent former child welfare clients. Eur Child Adolesc Psychiatry. 2013;22(6):349-355. doi:10.1007/s00787-012-0372-8 23296473

[5] Brännström L, Forsman H, Vinnerljung B, Almquist YB The truly disadvantaged? midlife outcome dynamics of individuals with experiences of out-of-home care. Child Abuse Negl. 2017;67:408-418. doi:10.1016/j.chiabu.2016.11.009 27884505

[6] Brännström L, Vinnerljung B, Forsman H, Almquist YB Children placed in out-of-home care as midlife adults: are they still disadvantaged or have they caught up with their peers? Child Maltreat. 2017;22(3):205-214. doi:10.1177/1077559517701855 28378598 PMC5497936

[7] Zlotnick C, Tam TW, Soman LA Life course outcomes on mental and physical health: the impact of foster care on adulthood. Am J Public Health. 2012;102(3):534-540. doi:10.2105/AJPH.2011.300285 22390519 PMC3487656

[8] Behr Gomes Jardim G, Novelo M, Spanemberg L, Influence of childhood abuse and neglect subtypes on late-life suicide risk beyond depression. Child Abuse Negl. 2018;80:249-256. doi:10.1016/j.chiabu.2018.03.029 29631256

[9] Choi NG, DiNitto DM, Marti CN, Segal SP Adverse childhood experiences and suicide attempts among those with mental and substance use disorders. Child Abuse Negl. 2017;69:252-262. doi:10.1016/j.chiabu.2017.04.024 28500922

[10] Bruffaerts R, Demyttenaere K, Borges G, Childhood adversities as risk factors for onset and persistence of suicidal behaviour. Br J Psychiatry. 2010;197(1):20-27. doi:10.1192/bjp.bp.109.074716 20592429 PMC2894980

[11] Angelakis I, Gillespie EL, Panagioti M Childhood maltreatment and adult suicidality: a comprehensive systematic review with meta-analysis. Psychol Med. 2019;49(7):1057-1078. doi:10.1017/S0033291718003823 30608046 PMC6498789

[12] Enns MW, Cox BJ, Afifi TO, De Graaf R, Ten Have M, Sareen J Childhood adversities and risk for suicidal ideation and attempts: a longitudinal population-based study. Psychol Med. 2006;36(12):1769-1778. doi:10.1017/S0033291706008646 16999880

[13] Sachs-Ericsson NJ, Rushing NC, Stanley IH, Sheffler J In my end is my beginning: developmental trajectories of adverse childhood experiences to late-life suicide. Aging Ment Health. 2016;20(2):139-165. doi:10.1080/13607863.2015.1063107 26264208

[14] Goldston DB, Erkanli A, Daniel SS, Heilbron N, Weller BE, Doyle O Developmental trajectories of suicidal thoughts and behaviors from adolescence through adulthood. J Am Acad Child Adolesc Psychiatry. 2016;55(5):400-407.e1. doi:10.1016/j.jaac.2016.02.010 27126854 PMC5035543

[15] Séguin M, Beauchamp G, Robert M, DiMambro M, Turecki G Developmental model of suicide trajectories. Br J Psychiatry. 2014;205(2):120-126. doi:10.1192/bjp.bp.113.139949 24809398

[16] Sosial-og helsedirektoratet Nasjonale retningslinjer for forebygging av selvmord i psykisk helsevern Oslo. Accessed March 20, 2020. https://www.helsedirektoratet.no/retningslinjer/forebygging-av-selvmord-i-psykisk-helsevern/Forebygging%20av%20selvmord%20i%20psykisk%20helsevern%20%E2%80%93%20Nasjonal%20faglig%20retningslinje.pdf/_/attachment/inline/c55a5440-c10d-4b7e-a81e-b6d16a6cd8b3:f889797fc632d620ac4f98a1ce83db3208336927/Forebygging%20av%20selvmord%20i%20psykisk%20helsevern%20%E2%80%93%20Nasjonal%20faglig%20retningslinje.pdf

[17] Ystgaard M, Hestetun I, Loeb M, Mehlum L Is there a specific relationship between childhood sexual and physical abuse and repeated suicidal behavior? Child Abuse Negl. 2004;28(8):863-875. doi:10.1016/j.chiabu.2004.01.009 15350770

[18] Goldston DB, Daniel SS, Erkanli A, Suicide attempts in a longitudinal sample of adolescents followed through adulthood: evidence of escalation. J Consult Clin Psychol. 2015;83(2):253-264. doi:10.1037/a0038657 25622200 PMC4380814

[19] Fiske A, O’Riley AA Toward an understanding of late life suicidal behavior: the role of lifespan developmental theory. Aging Ment Health. 2016;20(2):123-130. doi:10.1080/13607863.2015.1078282 26305860

[20] Almquist YB, Grotta A, Vågerö D, Stenberg S-Å, Modin B Cohort profile update: the Stockholm birth cohort study. Int J Epidemiol. 2019;dyz185. doi:10.1093/ije/dyz185 31539027 PMC7266536

[21] Sundell K, Vinnerljung B, Löfholm CA, Humlesjö E Child protection in Stockholm: a local cohort study on childhood prevalence of investigations and service delivery. Child Youth Serv Rev. 2007;29(2):180-192. doi:10.1016/j.childyouth.2006.07.001

[22] Björkenstam C, Kosidou K, Björkenstam E Childhood adversity and risk of suicide: cohort study of 548 721 adolescents and young adults in Sweden. BMJ. 2017;357:j1334. doi:10.1136/bmj.j1334 28424157

[23] Björkenstam C Epidemiological studies of suicide: classification bias, drug use, and social circumstances. Accessed March 20, 2020. https://openarchive.ki.se/xmlui/handle/10616/41497

[24] Jackisch J, Brännström L, Almquist YB Troubled childhoods cast long shadows: childhood adversity and premature all-cause mortality in a Swedish cohort. SSM Popul Health. 2019;9:100506. doi:10.1016/j.ssmph.2019.100506 31720363 PMC6838963

[25] Almquist YB, Landstedt E, Jackisch J, Rajaleid K, Westerlund H, Hammarström A Prevailing over adversity: factors counteracting the long-term negative health influences of social and material disadvantages in youth. Int J Environ Res Public Health. 2018;15(9):1842. doi:10.3390/ijerph15091842 30150519 PMC6164040

[26] Wall-Wieler E, Almquist Y, Liu C, Vinnerljung B, Hjern A Intergenerational transmission of out-of-home care in Sweden: a population-based cohort study. Child Abuse Negl. 2018;83:42-51. doi:10.1016/j.chiabu.2018.07.007 30016744

[27] Franzén E, Vinnerljung B, Hjern A The epidemiology of out-of-home care for children and youth: a national cohort study. Br J Soc Work. 2008;38(6):1043-1059. doi:10.1093/bjsw/bcl380

[28] Nagin DS Group-Based Trajectory Modeling: an Overview. Handbook of Quantitative Criminology. Springer; 2010:53-67. 10.1007/s10940-010-9113-7 PMC2994902 21132047

[29] Nagin DS, Odgers CL Group-based trajectory modeling (nearly) two decades later. J Quant Criminol. 2010;26(4):445-453. doi:10.1007/s10940-010-9113-7 21132047 PMC2994902

[30] O’Connor RC, Nock MK The psychology of suicidal behaviour. Lancet Psychiatry. 2014;1(1):73-85. doi:10.1016/S2215-0366(14)70222-6 26360404

[31] Arnett JJ, Žukauskienė R, Sugimura K The new life stage of emerging adulthood at ages 18-29 years: implications for mental health. Lancet Psychiatry. 2014;1(7):569-576. doi:10.1016/S2215-0366(14)00080-7 26361316

[32] Osgood DW, Foster EM, Flanagan C, Ruth GR On Your Own Without a Net: the Transition to Adulthood for Vulnerable Populations. University of Chicago Press; 2005. doi:10.7208/chicago/9780226637853.001.0001

[33] Evenson RJ, Simon RW Clarifying the relationship between parenthood and depression. J Health Soc Behav. 2005;46(4):341-358. doi:10.1177/002214650504600403 16433280

[34] Bruskas D Children in foster care: a vulnerable population at risk. J Child Adolesc Psychiatr Nurs. 2008;21(2):70-77. doi:10.1111/j.1744-6171.2008.00134.x 18429837

[35] Johnson PR, Yoken C, Voss R Family foster care placement: the child’s perspective. Child Welfare. 1995;74(5):959.

[36] Vinnerljung B, Sallnäs M, Berlin M Placement breakdowns in long-term foster care–a regional Swedish study. Child Fam Soc Work. 2017;22(1):15-25. doi:10.1111/cfs.12189

[37] Goldsmith SK, Pellmar TC, Kleinman AM, Bunney WE Reducing Suicide: A National Imperative. National Academies Press; 2002. 25057611

[38] Gao M, Brännström L, Almquist YB Exposure to out-of-home care in childhood and adult all-cause mortality: a cohort study. Int J Epidemiol. 2017;46(3):1010-1017. 28031308 10.1093/ije/dyw295 PMC5837321

[39] Ribeiro JD, Franklin JC, Fox KR, Self-injurious thoughts and behaviors as risk factors for future suicide ideation, attempts, and death: a meta-analysis of longitudinal studies. Psychol Med. 2016;46(2):225-236. doi:10.1017/S0033291715001804 26370729 PMC4774896

[40] Ludvigsson JF, Andersson E, Ekbom A, External review and validation of the Swedish national inpatient register. BMC Public Health. 2011;11(1):450. doi:10.1186/1471-2458-11-450 21658213 PMC3142234

[41] Dube SR, Anda RF, Felitti VJ, Chapman DP, Williamson DF, Giles WH Childhood abuse, household dysfunction, and the risk of attempted suicide throughout the life span: findings from the Adverse Childhood Experiences Study. JAMA. 2001;286(24):3089-3096. doi:10.1001/jama.286.24.3089 11754674

[42] Gallagher CA, Dobrin A Can juvenile justice detention facilities meet the call of the American Academy of Pediatrics and National Commission on Correctional Health Care? a national analysis of current practices. Pediatrics. 2007;119(4):e991-e1001. doi:10.1542/peds.2006-0959 17403835

[43] Butterworth S, Singh SP, Birchwood M, Transitioning care-leavers with mental health needs: “they set you up to fail!”. Child Adolesc Ment Health. 2017;22(3):138-147. doi:10.1111/camh.12171 32680381

[44] Vinnerljung B, Brännström L, Hjern A Disability pension among adult former child welfare clients: a Swedish national cohort study. Child Youth Serv Rev. 2015;56:169-176. doi:10.1016/j.childyouth.2015.07.001

[45] Mann JJ, Apter A, Bertolote J, Suicide prevention strategies: a systematic review. JAMA. 2005;294(16):2064-2074. doi:10.1001/jama.294.16.2064 16249421

[46] Zalsman G, Hawton K, Wasserman D, Suicide prevention strategies revisited: 10-year systematic review. Lancet Psychiatry. 2016;3(7):646-659. doi:10.1016/S2215-0366(16)30030-X 27289303

