# Supplementary material for: Association of Child Placement in Out-of-Home Care With Trajectories of Hospitalization Because of Suicide Attempts From Early to Late Adulthood
Source: JAMA Netw Open. 2020 Jun 2;3(6):e206639. doi: 10.1001/jamanetworkopen.2020.6639 (PMC7267851; doi:10.1001/jamanetworkopen.2020.6639)
Supplement: Supplement. — eTable 1. Percentage Distribution and Mean Number of Hospitalizations Among Individuals With at Least One Hospitalization Due to Suicide Attempts, Across the Study Variables and Trajectories eTable 2. Model Fit Statistics for the Group-Based Trajectory Modelling (n=525) eTable 3. Fitted Coefficients (Maximum Likelihood Estimates) for the Preferred Model (Logit Model With Four Trajectories and a Cubic Polynomial Function), Derived From Group-Based Trajectory Modelling (n=525) eTable 4. Distribution of Suicide Across Trajectories of Hospitalization Due to Suicide Attempts (n=14,559) eFigure. Distribution (%) of the Number of Hospitalizations Due to Suicide Attempts Across the Trajectories (n=525) [file jamanetwopen-3-e206639-s001.pdf]

Supplementary Online Content

Almquist YB, Rojas Y, Vinnerljung B, Brännström L. Association of child placement in out-of-home care with trajectories of hospitalization because of suicide attempts from early to late adulthood. *JAMA Netw Open*. 2020;3(6):e206639. doi:10.1001/jamanetworkopen.2020.6639

eTable 1. Percentage Distribution and Mean Number of Hospitalizations Among Individuals With at Least One Hospitalization Due to Suicide Attempts, Across the Study Variables and Trajectories

eTable 2. Model Fit Statistics for the Group-Based Trajectory Modelling (n=525)

eTable 3. Fitted Coefficients (Maximum Likelihood Estimates) for the Preferred Model (Logit Model With Four Trajectories and a Cubic Polynomial Function), Derived From Group-Based Trajectory Modelling (n=525)

eTable 4. Distribution of Suicide Across Trajectories of Hospitalization Due to Suicide Attempts (n=14,559)

eFigure. Distribution (%) of the Number of Hospitalizations Due to Suicide Attempts Across the Trajectories (n=525)

This supplementary material has been provided by the authors to give readers additional information about their work.

© 2020 Alquist YB et al. *JAMA Network Open.*

eTable 1. Percentage distribution and mean number of hospitalizations among individuals with at least one hospitalization due to suicide attempts, across the study variables and trajectories.

	Percentage (%) with at least one hospitalization due to suicide attempts[1]	Mean number of hospitalizations among individuals with at least one hospitalization due to suicide attempts[2]	Percentage (%) individuals in Trajectory 1: Peak in middle adulthood[2]	Percentage (%) individuals in Trajectory 2: Stable low across adulthood[2]	Percentage (%) individuals in Trajectory 3: Peak in young adulthood[2]	Percentage (%) individuals in Trajectory 4: Peak in emerging adulthood[2]
	3.6	2.04	12.6	31.8	40.0	15.6
**Variables**						
**Out-of-home care**						
Yes	9.9	2.24	10.7	29.0	45.8	14.5
No	3.0	1.97	13.2	32.7	38.1	16.0
**Sex**						
Women	3.7	2.09	14.0	33.0	36.0	17.1
Men	3.5	1.98	11.1	30.7	44.1	14.2
**Social class**						
High	3.0	2.09	13.5	32.7	40.8	13.0
Low	4.2	2.00	11.9	31.1	39.4	17.8
**Educational level**						
High	2.8	2.18	12.4	29.5	42.9	15.2
Low	3.9	2.06	12.6	32.4	39.3	15.7
**Poverty**						
Yes	7.0	2.04	11.8	29.4	44.7	14.1
No	2.9	2.03	13.0	33.0	37.8	16.3
**Death**						
Yes	5.0	1.96	11.1	28.9	42.2	17.8
No	3.5	2.04	12.7	32.1	39.8	15.4
**Criminality**						
Yes	6.8	2.23	12.8	32.1	42.3	12.8
No	3.3	2.00	12.5	31.8	39.6	16.1
**Alcohol misuse**						
Yes	7.9	2.01	5.6	26.8	49.3	18.3
No	3.3	2.04	13.7	32.6	38.6	15.2
**Mental health problems**						
Yes	8.4	2.17	10.3	25.6	47.4	16.7
No	3.3	2.01	13.0	32.9	38.7	15.4

[1] n=14,559; [2] n=525

© 2020 Alquist YB et al. *JAMA Network Open.*

eTable 2. Model fit statistics for the group-based trajectory modelling (n=525).

	**Nonsymmetric or highly singular variance matrix**	**Small groups (n<5%)**	**Bayesian Information Criterion (BIC)***
**Polynomial type**			
Intercept			
2 trajectories	No	Yes	-3461.28
3 trajectories	No	Yes	-3467.54
4 trajectories	No	Yes	-3473.80
5 trajectories	Yes	Yes	-3480.07
6 trajectories	Yes	Yes	-3486.33
7 trajectories	Yes	Yes	-3492.59
Linear			
2 trajectories	No	Yes	-3460.78
3 trajectories	No	Yes	-3456.84
4 trajectories	Yes	Yes	-3482.63
5 trajectories	Yes	Yes	-3472.01
6 trajectories	No	Yes	-3478.79
7 trajectories	Yes	Yes	-3492.20
Quadratic			
2 trajectories	**No**	**No**	**-3452.72**
3 trajectories	No	Yes	-3443.17
4 trajectories	No	Yes	-3435.70
5 trajectories	No	Yes	-3438.88
6 trajectories	No	Yes	-3447.09
7 trajectories	No	Yes	-3446.46
Cubic			
2 trajectories	**No**	**No**	**-3453.10**
3 trajectories	**No**	**No**	**-3451.19**
4 trajectories[a]	**No**	**No**	**-3458.17**
5 trajectories	No	Yes	-3458.11
6 trajectories	No	Yes	-3455.45
7 trajectories	Yes	Yes	-3461.80

* The lower the BIC, the better the model fit.

[a] Preferred model. Model with nonsymmetric or highly singular variance matrix were disqualified, as were models with one or more small groups (n<5%). Thus, remaining models were: the two-trajectory model with quadratic polynomial, as well as the two-, three-, and four-trajectory models with cubic polynomial. Of these, the four-trajectory model had the lowest BIC and was thus chosen.

© 2020 Alquist YB et al. *JAMA Network Open.*

eTable 3. Fitted coefficients (maximum likelihood estimates) for the preferred model (logit model with four trajectories and a cubic polynomial function), derived from group-based trajectory modelling (n=525).

**Trajectory/ Group**	**Parameter**	**Estimate**	**Standard error**	**T for H0: Parameter=0**	**Prob > |T|**
1					
	Intercept	-7.02994	94.02447	-0.075	0.9404
	Linear	0.23768	11.60894	0.020	0.9837
	Quadratic	0.01555	0.47375	0.033	0.9738
	Cubic	-0.00068	0.00639	-0.107	0.9151
2					
	Intercept	-6.86354	10.41104	-0.659	0.5097
	Linear	-0.15216	0.97813	-0.156	0.8764
	Quadratic	0.02152	0.03016	0.713	0.4757
	Cubic	-0.00068	0.00031	-1.230	0.2187
3					
	Intercept	-2.32132	3.96600	-0.585	0.5583
	Linear	-0.10704	0.31857	-0.336	0.7369
	Quadratic	0.00294	0.00793	0.370	0.7112
	Cubic	-0.00002	0.00006	-0.340	0.7339
4					
	Intercept	-132.59235	111.02507	-1.194	0.2324
	Linear	7.07719	6.62292	1.069	0.2853
	Quadratic	-0.12435	0.13080	-0.951	0.3418
	Cubic	0.00070	0.00086	0.819	0.4125
**Group membership**[a]					
1	%	12.13288	2.54780	4.762	0.0000
2	%	32.38007	4.33939	7.462	0.0000
3	%	41.22317	7.13070	5.781	0.0000
4	%	14.26388	5.20628	2.740	0.0062

[a] The percentages are based on *predicted* group membership and do therefore do not exactly correspond to the percentages presented in the manuscript for the same groups.

© 2020 Alquist YB et al. *JAMA Network Open.*

eTable 4. Distribution of suicide across trajectories of hospitalization due to suicide attempts (n=14,559).

	**Suicide**	
	**n**	**%**
	154	1.1
**Variables**		
**Hospitalization due to suicide attempts**		
No	107	0.8
Yes	47	9.0
**Trajectories of hospitalization due to suicide attempts**		
Trajectory 1: Peak in middle adulthood	8	12.1
Trajectory 2: Stable low across adulthood	9	5.4
Trajectory 3: Peak in young adulthood	23	11.0
Trajectory 4: Peak in emerging adulthood	7	8.5

The coding of suicide (ages 20-65) is based on the same (underlying) ICD diagnoses as for suicide attempts. Information based on death records in the Cause of Death Register.

© 2020 Alquist YB et al. *JAMA Network Open.*

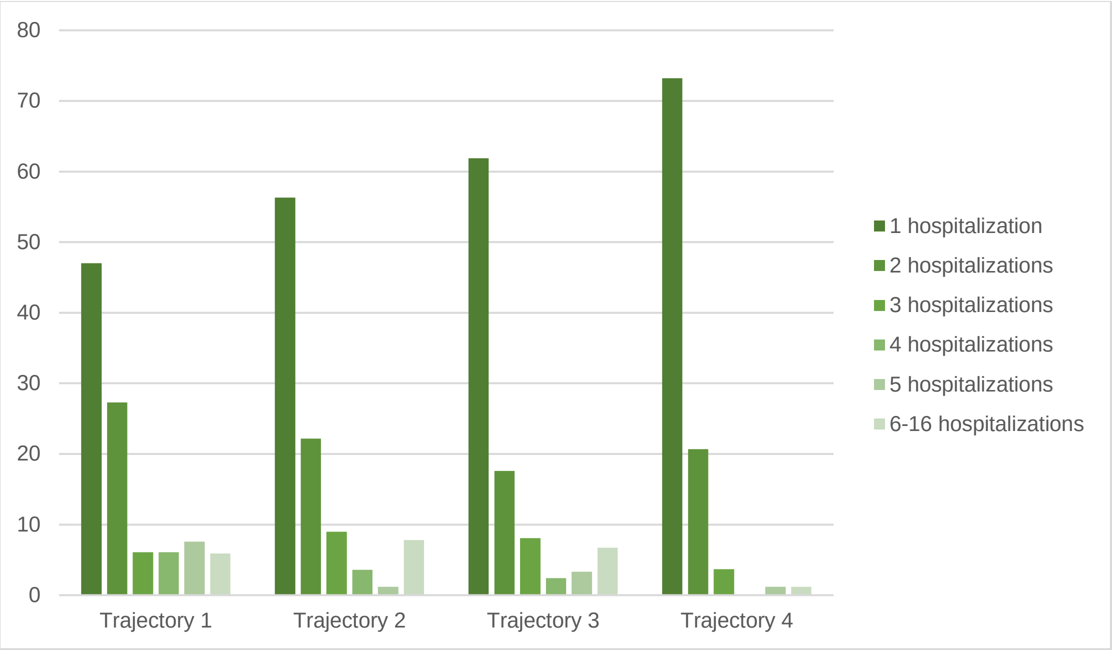

eFigure 1. Distribution (%) of the number of hospitalizations due to suicide attempts across the trajectories (n=525). Mean number of hospitalizations: Trajectory 1=2.30; Trajectory 2=2.20; Trajectory 3=2.08; Trajectory 4=1.39.

© 2020 Alquist YB et al. *JAMA Network Open.*
